# Towards the ecological automated measurement of joint attention: Development of an interactive eye-tracking battery for joint attention in children with and without autism

**DOI:** 10.3758/s13428-026-03017-w

**Published:** 2026-05-13

**Authors:** Christy D. Yoon, Hedda Meadan, Frederick Shic

**Affiliations:** 1 Waisman Center, University of Wisconsin-Madison, Madison, WI USA; 2 Department of Special Education and Child Development, University of North Carolina at Charlotte, Charlotte, NC USA; 3 Center for Child Health, Behavior and Development, Seattle Children’s Research Institute, Seattle, WA USA; 4 Department of Pediatrics, University of Washington School of Medicine, Seattle, WA USA

**Keywords:** autism, eye tracking, ecological validity, gaze contingency, iterative approach, joint attention, team science

## Abstract

Eye tracking has emerged as a powerful tool for advancing autism research, including diagnostics and interventions. However, studies on joint attention (JA) in autistic children have predominantly concentrated on the responding to joint attention (RJA) construct, with limited focus on the initiating joint attention (IJA) construct. Moreover, despite the interactive nature of JA, researchers have often relied on passive paradigms to study JA in this population. To address these gaps, we developed a gaze-contingent eye-tracking battery targeting three developmentally appropriate JA skills for young children: RJA, IJA to request, and IJA to comment or reference. The development process was multifaceted and iterative, involving a series of collaborative steps and the allocation of various resources. These steps included determining the motion format and stimulus type, designing and prototyping the stimuli, recruiting an actor to serve as a communication partner in the stimuli, recording and editing videos for the stimuli, and building and test-running the battery. We developed the Interactive Eye Tracking for Joint Attention (IET-JA) battery, which consists of 32 JA stimuli: 16 RJA, 8 IJA-Request, and 8 IJA-Comment/Reference. The stimuli are dynamic (i.e., videos) and feature a preprogrammed interactive human communication partner who is responsive to the participant’s gaze. The IET-JA takes approximately 8 minutes to complete, and its duration is expected to vary based on the participant’s level of engagement. Implications for advancing methodologies, fostering team science, and enhancing iterative processes are discussed.

**Supplementary Information:**

The online version contains supplementary material available at 10.3758/s13428-026-03017-w.

## Introduction

Eye tracking is a powerful tool that provides a direct window into visual perception and attention by allowing highly precise quantification of the spatial and temporal gaze patterns of individuals. Eye tracking entails many advantages that advance it as a potent tool for studying autistic children. Some key advantages include non-invasiveness, objectivity, easy implementation, high temporal and spatial resolution, ability to capture subconscious and involuntary eye movements, and optimality to a wide age range and ability level (Dawson et al., 2012; Karatekin, 2007; Pierce et al., 2016; Shic, 2013; Shic et al., 2022; Zoccolan et al., 2010).

Since 2002 (Klin et al., 2002; Pelphrey et al., 2002; van Der Geest et al., 2002a, 2002b), autism research has seen growing momentum in the use of eye tracking to uncover nuanced aspects of autism and advance diagnostic (e.g., Jones et al., 2023; Pierce et al., 2016), predictive (e.g., Hendry et al., 2018; Shic et al., 2020), prognostic (e.g., Bacon et al., 2020), and treatment response (e.g., Bradshaw et al., 2019; Robain et al., 2020; Yoon et al., 2025a; Zhao et al., 2022) biomarkers of autism. In particular, eye-tracking research efforts have intensified in quantifying the components of social attention (i.e., attention to social elements within the displayed stimulus; e.g., Chawarska et al., 2016; Elsabbagh et al., 2014; Jones et al., 2008; Latrèche et al., 2021; Murias et al., 2018; Shic et al., 2020, 2023), with joint attention (JA) being a salient facet (Dawson et al., 2012; Salley & Colombo, 2016).

JA is a fundamental aspect of social and cognitive development that functions to gain, maintain, and shift shared attention with a communication partner (Dawson et al., 2004; Mundy, 2016, 2018; Vivanti et al., 2017). It manifests in two constructs that capture the reciprocal nature of JA interactions and form the foundation for effective social communication development: responding to joint attention (RJA), the ability to respond, usually with gaze, to social bids; and initiating joint attention (IJA), the ability to direct a communication partner’s attention to a shared interest to either request (protoimperative) or comment or reference (protodeclarative; Bakeman & Adamson, 1984; Bottema-Beutel, 2016; Meindl & Cannella-Malone, 2011; Mundy, 2018; Mundy & Newell, 2007; White et al., 2011). The study of JA is of paramount importance due to its pivotal role in characterizing autism (American Psychiatric Association, 2013). Reduced JA is a hallmark feature of autism that is often evident from an early age and serves as a window into social communication differences in autistic children (Charman, 2003; Chiang et al., 2008; Dawson et al., 2004; Lord et al., 2000; Mundy, 2016; Nyström et al., 2019).

As such, recent years have seen increased recognition of eye tracking as a means of quantifying JA in autistic children. These studies, however, have focused heavily on the RJA construct (Yoon et al., 2026), resulting in contradicting findings (e.g., Cilia et al., 2022; de Belen et al., 2023; Falck-Ytter et al., 2012, 2015; Swanson & Siller, 2013; Zhang et al., 2022). For example, while Falck-Ytter and colleagues (2015) and Swanson and Siller found no significant group differences in gaze-following accuracy and gaze allocation, respectively, between autistic and typically developing children, Falck-Ytter and colleagues (2012) and de Belen and colleagues revealed a significant group difference in gaze-following accuracy between the two groups (i.e., autistic children performed fewer and slower gaze-following movements compared to their typically developing peers).

A commonly reported factor perceived to be responsible for heterogeneity in RJA findings is the nature of the stimuli (Billeci et al., 2016, 2017; Zhang et al., 2022). Several researchers examined and revealed the impact of the ecological nature of social stimuli (i.e., dynamic, video-based moving action formats vs. static, still images) on gaze patterns in autistic children (Chevallier et al., 2015; Cilia et al., 2019; Saitovitch et al., 2013; Shic et al., 2014; Speer et al., 2007). Chevallier and colleagues (2015) compared three classes of stimuli (photos, videos of faces and objects presented side by side, and videos of children playing with objects in a naturalistic context), in which the video stimuli featuring children playing emerged as a particularly sensitive tool for distinguishing between autistic and typically developing children. Cilia and colleagues (2019) compared two classes of stimuli (photos and videos); their findings indicated that both groups showed similar gaze patterns during static stimuli, whereas autistic children showed distinct gaze patterns toward typically developing peers during dynamic stimuli. Saitovitch and colleagues (2013) administered four classes of stimuli (video with human actors, video with cartoons, photo with human actors, and photo with cartoons); they revealed significantly reduced fixations on eyes and faces when viewing human actors compared to cartoons, videos compared to photos, and video with human actors compared to photos with human actors. Shic and colleagues (2014) administered three classes of stimuli (photo of the face of a human actor, video with a smiling face of a human actor, and video with a human actor providing motherese) and found that infants later developing autism exhibited less attention to the informative inner areas of the face during the video of the actor providing speech. Lastly, Speer and colleagues (2007) compared four classes of stimuli (social video, social photo, isolated video, and isolated photo) and found differences between children with and without autism only during social video. There is also evidence from animal studies (Blatter & Schultz, 2006) and functional neuroimaging data (Weisberg et al., 2014) that corroborate dynamic stimuli as more potent than static stimuli in eliciting differences between individuals with autism and controls.

It is important to recognize that static and dynamic stimuli can have distinct advantages: for instance, static stimuli can enable enhanced experimental control to minimize variability in the constructs under investigation, whereas dynamic stimuli can emulate more naturalistic, real-world phenomena. However, findings from five studies (Chevallier et al., 2015; Cilia et al., 2019; Saitovitch et al., 2013; Shic et al., 2014; Speer et al., 2007) suggest that the ecological relevance of social stimuli significantly increases the effectiveness of eye-tracking stimuli in capturing distinct gaze patterns among autistic children.

JA is, by its nature, interactive and dynamic. Because the ecological validity of stimuli appears to impact the ability of eye-tracking methodologies to elicit social attention differences between individuals with autism and other groups more robustly, it can be expected that a more optimal eye-tracking JA task would involve stimuli that incorporate dynamic interactivity. Gaze-contingent stimuli, which react (adapt or change) based on gaze allocation on the displayed stimulus (Just & Carpenter, 1976), embody the dynamic interactivity crucial for studying JA. For example, if a person fixates on a specific area on the screen, a visual element might be triggered to change or move depending on the task. The key characteristic of gaze contingency that renders gaze-contingent stimuli critical for JA research is establishing the communication partner featured in the stimuli as a responsive entity. This dynamic interaction simulates more realistic and natural JA scenarios by having the communication partner respond to the participant’s gaze, thereby augmenting ecological relevance. Moreover, the integration of gaze contingency offers a high level of experimental control and flexibility, allowing for precise manipulation of timing and a systematic examination of JA skills. Despite such advantages, however, there has been limited utilization of gaze-contingent stimuli in investigating interactive JA behaviors. To our knowledge, only two eye-tracking studies (Liu et al., 2024; Wang, Hoi et al., 2020) have utilized gaze-contingent stimuli to examine JA in autistic children (Yoon et al., 2026). Specifically, Liu and colleagues employed this method in a virtual game format to investigate how different cues from a virtual character influence the RJA performance of autistic children and found that various cue types impacted the game scores of autistic children, with no significant effect observed in typically developing children. Similarly, Wang, Hoi, and colleagues employed this method to explore reciprocal JA between a virtual character and autistic children and found significant differences in sensitivity to virtual faces following gaze-leading in JA stimuli, as well as differing effects of JA on subsequent object-looking behaviors between autistic and typically developing children. However, the impact of human versus anthropomorphic (e.g., cartoon) agents on gaze behaviors has been observed across various groups (Riby & Hancock, 2009; Saitovitch et al., 2013), and on facial emotion recognition in non-eye-tracking studies (Atherton & Cross, 2022; Brosnan et al., 2015). Thus, we recognized that further exploration of gaze-contingent stimuli with a human communication partner could contribute to the literature.

Therefore, this study aimed to develop a gaze-contingent eye-tracking battery for JA (hereinafter referred to as the Interactive Eye Tracking for Joint Attention, IET-JA), focusing on three developmentally appropriate JA skills for young children: RJA, IJA to request, and IJA to comment or reference. This development sought to enhance the ecological relevance of current eye-tracking stimuli for studying JA in autistic children while maximizing experimental control to distinguish gaze behaviors uniquely associated with different JA constructs.

## Methods

**Fig. 1:**
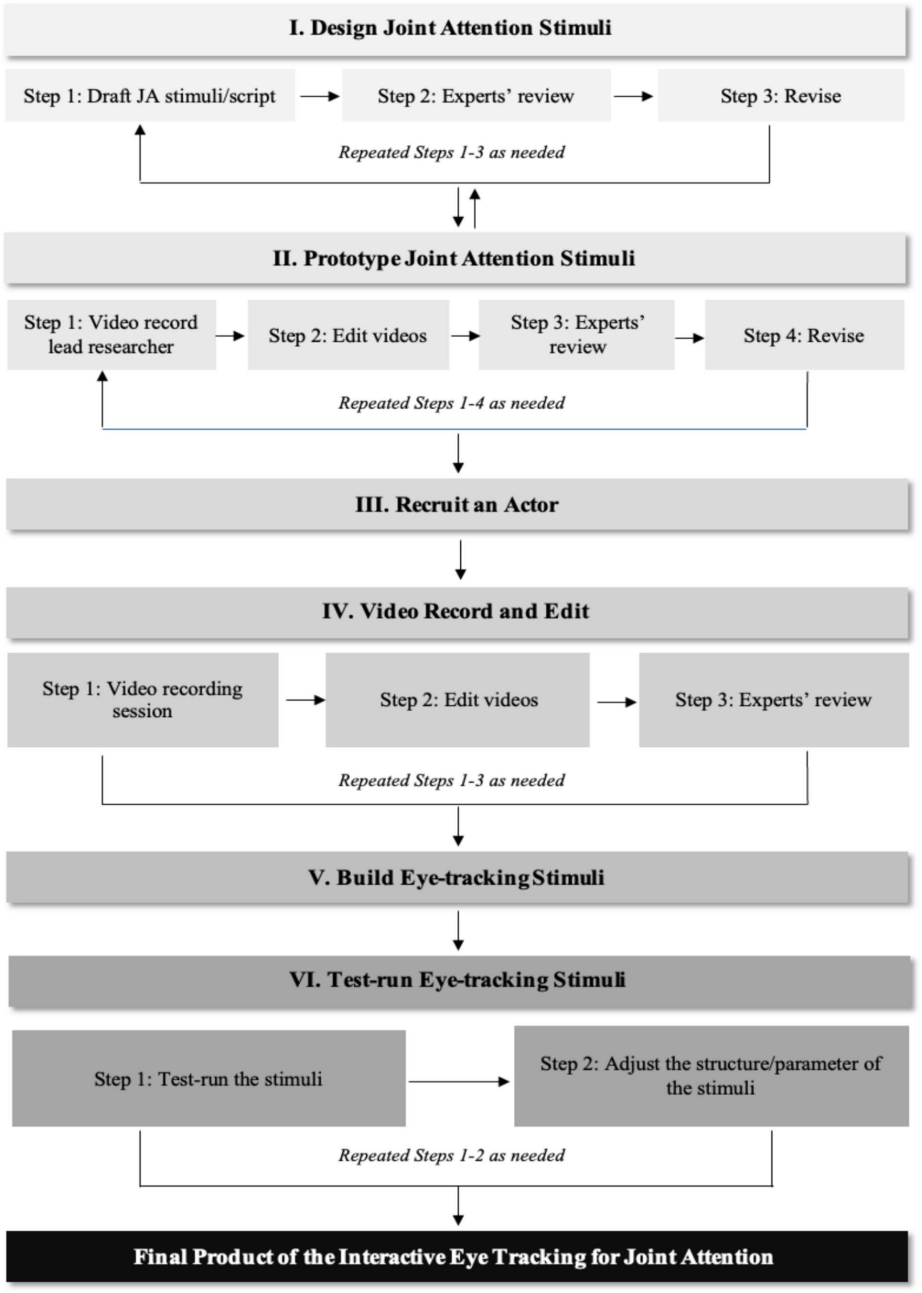
The iterative process for developing the Interactive Eye Tracking for Joint Attention (IET-JA) battery

The development of the IET-JA was a multifaceted and iterative process that involved a series of collaborative steps and resource allocation: (a) determining the motion format and type of stimuli; (b) designing and prototyping the stimuli for JA; (c) identifying and recruiting an actor to serve as a communication partner in the stimuli; (d) recording and editing the videos for the stimuli; and (e) building and conducting test runs of the stimuli. See Fig. 1 for a visual overview of the development process. The following sections detail the IET-JA development process.

### Stimuli motion format and type

Dynamic social stimuli (i.e., moving formats such as videos featuring human characters) were employed to develop an ecologically valid battery that reflects social nature. Additionally, the gaze-contingency approach (i.e., stimuli that respond to gaze) was integrated as a vital component of the battery to facilitate interactive JA episodes between the participant and the communication partner featured in the stimuli.

### Designing and prototyping joint attention stimuli

**Fig. 2:**
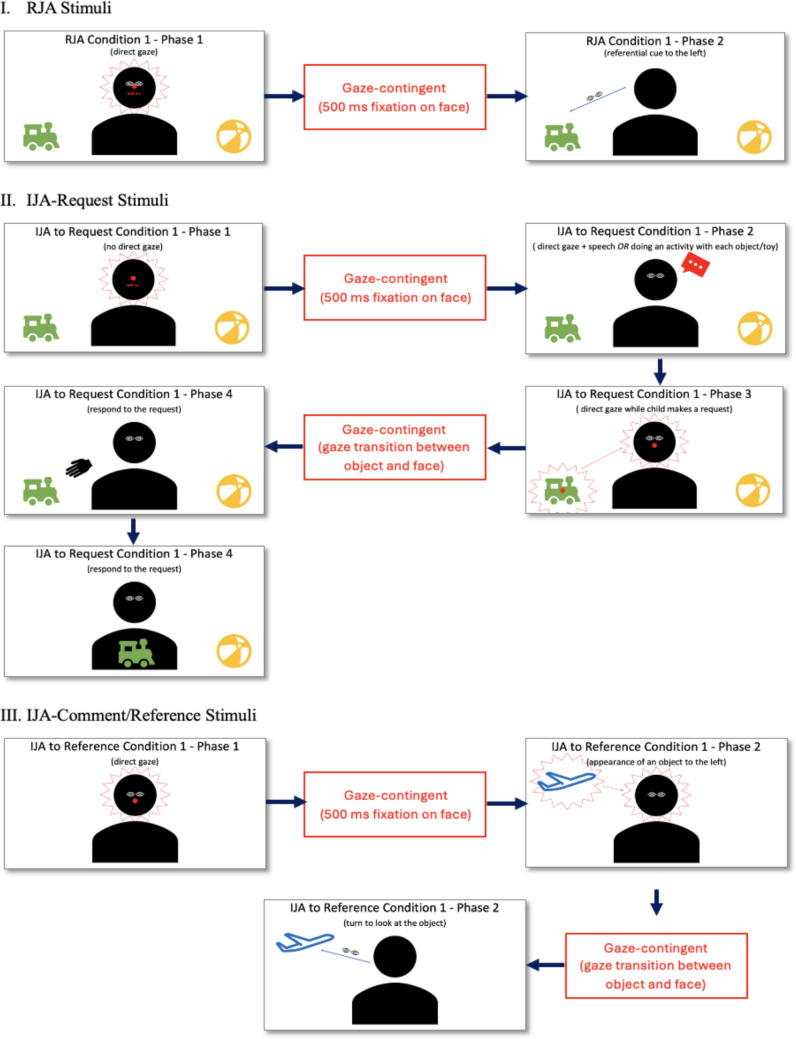
An example of a preliminary design for JA stimuli. *Note.* This figure presents an example of a preliminary design of JA stimuli that evolved during development and therefore differs from the final JA stimuli reported in the Results

**Fig. 3:**
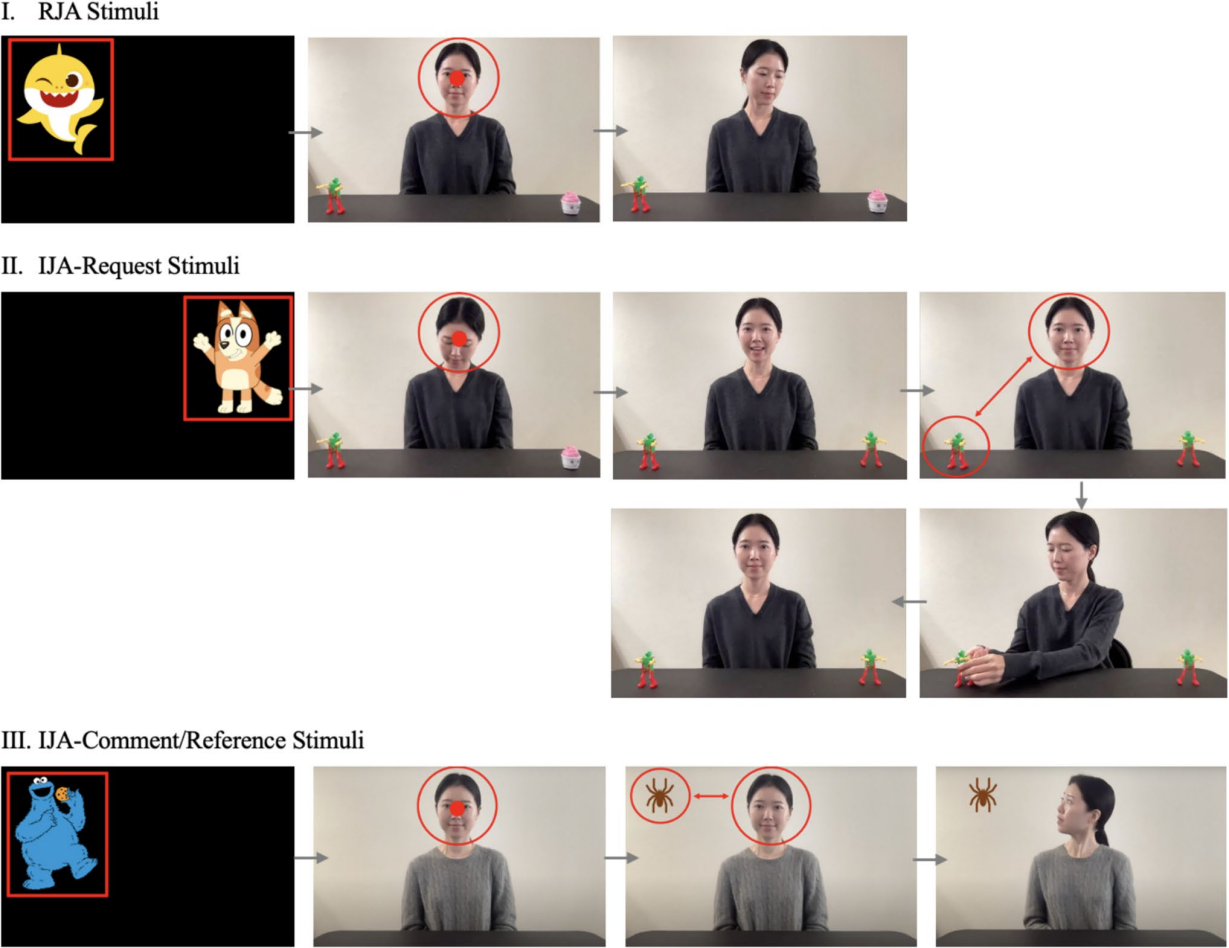
An example of a preliminary prototype for JA stimuli. *Note*. This figure presents an example of a preliminary prototype of JA stimuli that evolved during development and therefore differs from the final JA stimuli reported in the Results. *Red outlines* (i.e., arrow, circle, rectangle) indicate regions of interest and gaze behaviors relevant to gaze contingency (i.e., fixation on the face, alternating gaze between the face and an object)

The preliminary development of JA stimuli involved repeated sequences of designing, prototyping, and evaluating, all occurring concurrently in an interwoven manner (see Figs. 2 and 3 for an example of preliminary design and prototype for JA stimuli). The designing phase included identifying activities for JA and outlining scenarios and interactions between the child and the communication partner that correspond to the RJA and IJA stimuli, as detailed in the following sections. The feasibility and optimality of administering these stimuli using eye-tracking technology were considered during this phase. Experts reviewed the design to ensure it aligned with the study’s objectives and appropriateness. Following the expert review, the design of the stimuli entered the prototyping phase, which involved video-recording a researcher acting as a “pre-actress” to demonstrate the pre-approved design of the JA stimuli. This visual representation facilitated a practical evaluation of the stimuli’s feasibility and clarity. The designing and prototyping processes underwent cycles of review and revision as needed (e.g., refining dialogues, selecting objects to feature in the stimuli, and determining stimulus duration) to ensure that the final stimuli accurately measured JA skills using the gaze-contingent paradigm.

#### Responding to joint attention stimuli

A range of cues was explored in eye-tracking studies examining RJA in autistic children, from communication partners using gaze shifts alone to combinations of gaze shifts with one or more explicit cues (i.e., head-turning, pointing, or verbalization) for RJA bids (Yoon et al., 2026). Some findings suggest that RJA differences between autistic children and control children are more pronounced when subtler, less explicit cues are used, particularly during gaze shifts alone, compared to when gaze shifts are paired with more explicit cues such as pointing (e.g., Cilia et al., 2022; Franchini et al., 2017; Liu et al., 2024). However, the evidence remains limited. Therefore, we designed RJA stimuli that vary in cue explicitness: one event in which the communication partner uses gaze shifts alone, and another event in which gaze shifts are paired with a more explicit cue, such as head-turning. This design facilitates the investigation of children’s RJA skills across varying levels of cues provided for RJA.

#### Initiating joint attention to request stimuli

For IJA-Request stimuli, the goal was to address the challenge of distinguishing between the two functions of IJA: to request (protoimperative) and to comment or reference (protodeclarative). We operationalized the context of IJA to request as a scenario where the communication partner is aware of and has access to objects the child may desire to interact with or have activated (i.e., objects in the communication partner’s hand or located closer to the communication partner than the child; Whalen & Schreibman, 2003).

The key design choice was to use two tablets that display short videos, serving as ecological surrogates for real-world items a child may desire to “obtain.” In naturalistic settings, children’s requests often relate to tangible items or events initiated by others, such as opening a container to reveal its contents. However, these scenarios require considerable physical setup and rely on assumptions about the child’s ability to predict and anticipate the outcomes of such interactions. In contrast, using tablets capitalizes on children’s early understanding of screens as media that can “activate” to show desired content. Videos provide a natural and compelling substitute for tangible items, allowing the child to request something they want to “see”—an action that aligns with their expectation of screens as sources of dynamic and engaging stimuli. Additionally, including two tablets facilitates the expression of preferences, creating a situation where the child’s communicative intent can be observed clearly in a controlled manner.

Moreover, we designed a training trial for IJA-Request stimuli as an introductory component of the IET-JA to ensure the child understands the functional context of IJA-Request stimuli. This trial sought to convey that the videos featured on the tablets were not static images but would play upon request. Establishing this cognitive and contextual framework for IJA-Request stimuli was essential, akin to setting up a premise or solving a puzzle. Specifically, the training trial demonstrates the interaction mechanics (e.g., a request activates the video) while helping the child anticipate the outcomes of their communicative actions. This design ensures that the child recognizes the communication partner as a responsive agent (i.e., enabling video playback) and establishes the relevance of their request to achieving the desired outcome.

#### Initiating joint attention to comment/reference stimuli

The IJA function to comment or reference involves using nonverbal or verbal communication to share attention with a communication partner toward an object or event of interest, often when the partner is unaware of the stimulus. This function contrasts with the function to request, which often involves directing the partner’s attention to a desired object or event mutually recognized by both parties. Thus, we operationalized the context of IJA-Comment/Reference stimuli as a scenario in which a sudden, unexpected moving object—such as a crawling spider or a flying bird—enters the scene, capturing the child’s interest and motivating them to direct the communication partner’s attention to it. This approach aligns with the theoretical framework of IJA, which views commenting or referencing as a form of shared engagement and social referencing purely for social purposes (Bates et al., 1975; Bruinsma et al., 2004; Jones & Carr, 2004; Yoder et al., 2009), distinct from the request function. By constructing scenarios in which the communication partner is initially unaware of the object, IJA-Comment/Reference stimuli emphasize their spontaneous and referential nature.

#### Initial phase for joint attention stimuli

After several iterations of designing and prototyping JA stimuli, we included an initial phase for all stimuli in which the communication partner begins by looking down (e.g., Billeci et al., 2016, 2017), deliberately avoiding eye contact (i.e., non-dyadic) with the child and maintaining a downward gaze until the child initiates attention toward the communication partner. Implementing this initial phase uniformly across all stimuli establishes a neutral baseline from which the child’s spontaneous JA skills can be observed without influence from the communication partner. Accordingly, it maximizes the opportunity for a more precise assessment of the child’s natural inclination to seek JA interaction.

#### Attention-getter

In gaze-contingent experiments, in which stimuli respond to the participant’s gaze, it is crucial to establish control over the participant’s initial point of attention. Without this control, unintended triggering of experimental events may occur, which could undermine the reliability of the outcomes. To address this challenge, we implemented an attention-getter mechanism before the initial phase of all stimuli: a blank screen featuring an image, such as a star. This preparatory step is designed to direct the child’s attention to the screen and to ensure a consistent and controlled starting point for all stimuli. If necessary, prompts may be provided during this phase to help the child attend to the stimuli without these prompts influencing the evaluation of the child’s spontaneous JA skills being measured. This approach helps regulate the child’s readiness and contributes to the reliability and clarity of the task outcomes.

### Identifying and recruiting an actor

As discussed in the introduction, researchers indicate the impact of featuring human actors versus anthropomorphic agents on gaze behavior and facial emotion recognition. Specifically, more pronounced group differences were observed when viewing stimuli featuring human agents compared to when viewing those featuring animated agents (Atherton & Cross, 2022; Brosnan et al., 2015; Riby & Hancock, 2009; Saitovitch et al., 2013). Moreover, JA challenges are most relevant in the context of real-world interactions, which align more closely with human actors (Carter et al., 2014). While evidence suggests that nonhuman characters can be more effective at engaging autistic children (Meng et al., 2023), such engagement may alter the nature of the JA attentional process. Therefore, a human actor was recruited as the social element (i.e., the interactive communication partner in the stimuli) rather than creating animated characters.

#### Eligibility

There were a few eligibility criteria to be an actor in the stimuli. An actor needed to (a) be at least 18 years old, (b) be a fluent English speaker, and (c) have a neutral or conventional appearance (e.g., no visible tattoos or facial piercings). The adult requirement ensures that the actor understands and follows complex instructions and scripts. Identifying an adult actor who meets these criteria is also more feasible, which helps efficiently execute the development process. Additionally, the evaluation or direct instruction of interactional skills typically occurs with an adult model (e.g., clinician, teacher, parent). Using an adult actor, we parallel key interactions that form the basis for early child development, such as the dyadic relationship between a child and an adult. The clarity and comprehensibility of the language used in the battery are also paramount. A fluent English speaker ensures that prompts and instructions are delivered clearly, which is essential for children to understand and respond appropriately. This also sets expectations for guidance, helping the child understand what is being said. Lastly, the requirement for a neutral appearance minimizes any unintended influence on children’s attention. For example, visible tattoos, distinctive hairstyles, or piercings could serve as novel or highly salient stimuli that might divert children’s focus from the primary task. By reducing such potential distractions, a more controlled environment for stimuli is established. As a result, an adult communication partner, identified as a White female, was recruited to serve as a communication partner in the stimuli (we acknowledge that this is a limitation and will revisit it in the Discussion). Since eye-tracking studies examining JA in autistic children primarily included one female adult as a communication partner (e.g., Bacon et al., 2020; Cilia et al., 2019; Falck-Ytter et al., 2012; Vivanti et al., 2017), and a few studies that included both male and female actors did not explore the influence of the actor’s sex on gaze behaviors (Billeci et al., 2017; Maes et al., 2021; Zhang et al., 2022), it was determined that the communication partner’s sex might not be a significant confounding factor. Hereinafter, the terms ‘actress’ and ‘communication partner’ will be used interchangeably.

### Video recording and editing

**Fig. 4:**
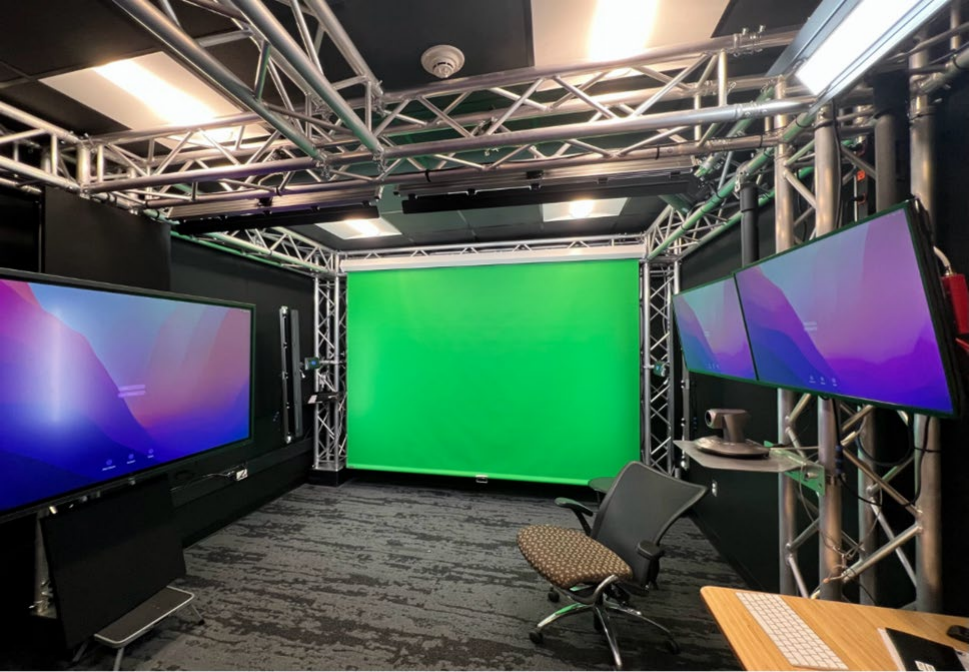
Photo of the studio where video recording sessions took place. *Note.* The studio is designed without windows to eliminate uncontrolled external light, and it is also equipped with soundproof materials to minimize external noise interference. The green-screen backdrop setup allows for versatile video production with custom backgrounds.

After recruiting the actress, video recording sessions took place over two days, each lasting three hours, in a professional studio at the University. The studio had no windows, eliminating uncontrolled external light sources, and it was equipped with soundproof materials (e.g., acoustic ceiling tiles and additional soundproof panels on the walls) to minimize external noise interference. See Fig. 4 for a photo of the studio where the recording sessions took place.

A digital specialist was present during both recording sessions to provide support and supervision. This role was crucial for maintaining the quality of the recordings by overseeing various technical aspects, including lighting, audio quality, camera angles, and output consistency (resolution: 1920 x 1080; frame rates: 29.97 fps; file format: MPEG-4; audio codec: AAC, Advanced Audio Codec; video codec: H.264, High-Efficiency Compression). Consistency in the recordings was especially important for the gaze-contingency element in developing the IET-JA. Gaze contingency requires mixing and matching different video segments based on the participant’s gaze behaviors. Because these segments are stitched together to create a seamless and cohesive experience, it was vital to ensure that no visible discrepancies in lighting, background characteristics, or audio quality were present. Such inconsistencies could draw participants’ attention to the scene’s composite nature, potentially leading them to focus on minor details rather than the intended content of the stimuli. Consistent start and end positions were employed to facilitate stitching, ensuring continuity across segments. These measures were essential for maintaining the integrity and validity of the experimental conditions, ensuring that participants’ attention remained on the primary stimuli rather than being diverted by irrelevant aspects of the presentation.

Approximately 100 videos were recorded during the first session and reviewed by experts who identified areas for improvement that could not be addressed through video editing: the actress’s visual appearance (e.g., removing eye makeup to enhance clarity in her gaze cues for RJA stimuli); content alignment within a phase (e.g., tablets intended to remain on were off during certain phases of IJA-Request stimuli); and the entrance of suddenly appearing objects for IJA-Comment/Reference stimuli. Consequently, a second recording session was held to implement these changes. Notably, the actress wore a neutral top intentionally selected for its lack of distinctive features or appeal; this decision was made to ensure a controlled environment by minimizing unnecessary distractions that could affect the child’s focus on the stimuli.

Approximately 100 additional videos were captured during the second recording session. These videos were edited in Adobe Premiere Pro, a video editing software, to enhance visuals and clarity. The edited videos were then reviewed by experts for the actress’s performance, clarity of communication, overall visual and auditory quality, and, most importantly, theoretical alignment with the JA behavioral construct under investigation. Based on feedback, necessary modifications were made to fine-tune the videos (e.g., masking, trimming, merging) to meet the study objectives. This cycle of expert review and video editing repeated until final approval was obtained from all involved experts, ensuring that the final products met the desired standards and were ready for integration into the eye-tracking battery.

### Building eye-tracking stimuli

**Fig. 5:**
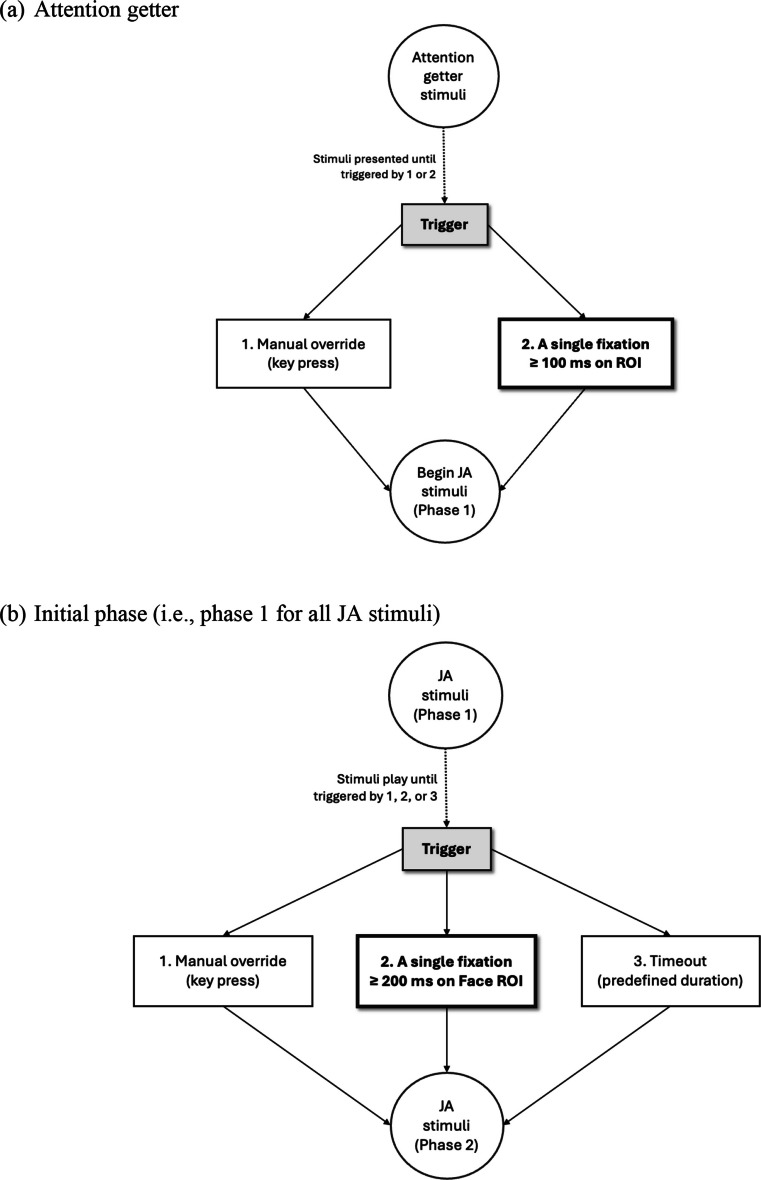

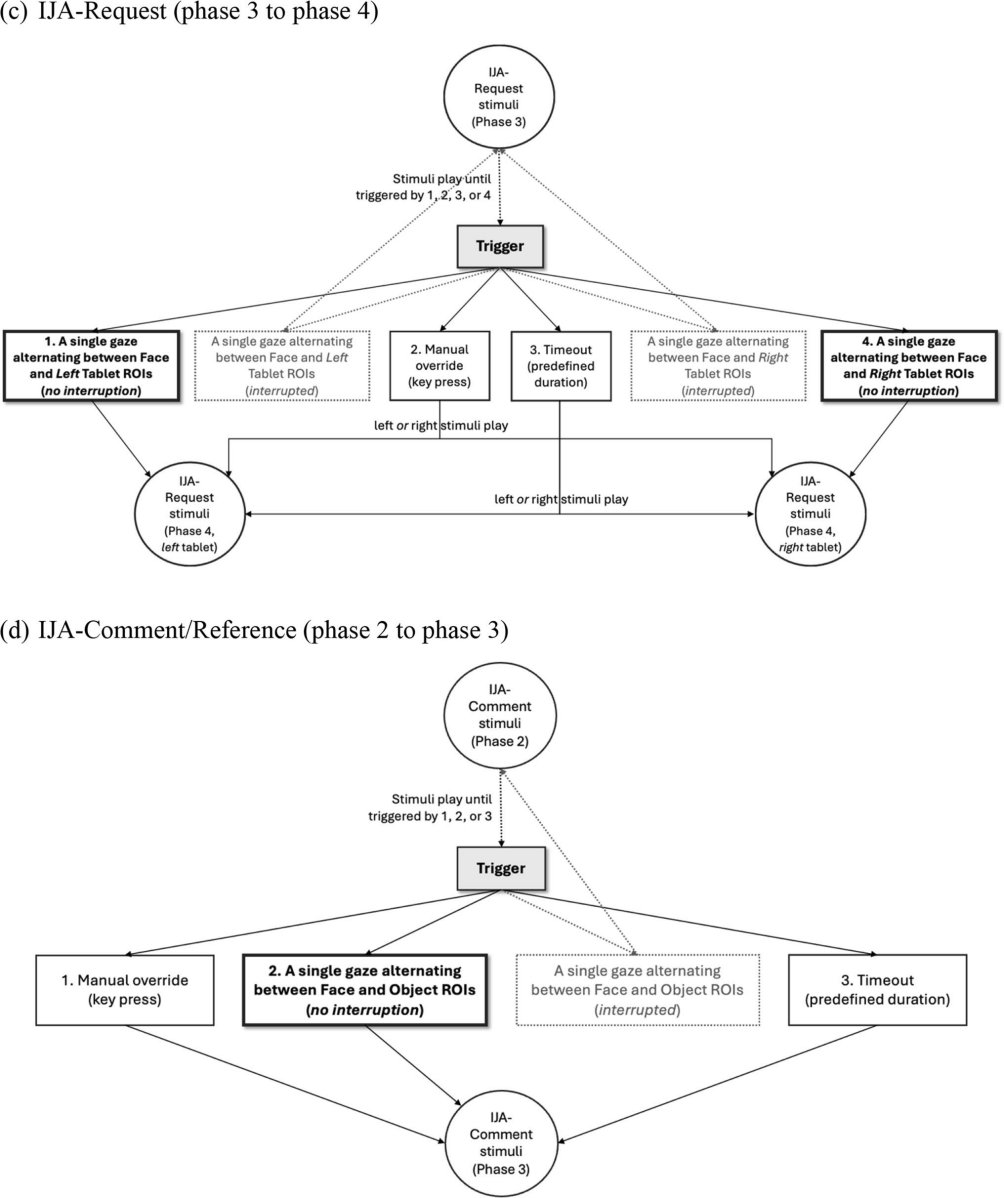
Conceptualization of the gaze-contingent experimental setup for phases involving interactions between the stimuli and the participant

The approved videos were prepared in formats compatible with the experiment delivery system. This process began with designing and coding the experimental flow into the selected delivery system. Several systems could have been utilized for this purpose, such as E-Prime, Neurobehavioral Systems for Presentation, or MATLAB Psychtoolbox. However, SR Research Experiment Builder (SR Research Experiment Builder 2.3.1 [Computer software], 2020), a graphical programming software for creating eye-tracking experiments, was chosen for its user-friendly interface, which allows researchers to visually design study elements. The video files were split into video and audio components to ensure compatibility with SR Research Builder. The video was saved in AVI container format using the Xvid codec (standard: MPEG-4 ASP; resolution: 1920 x 1080; frame rate: 30 Hz; bit rate: 12.0 Mbps), while the audio was extracted and saved in WAV format (sampling frequency: 44.1 kHz; bit depth: 16 bits). The stimuli were methodically configured using this interface. A critical aspect of this configuration involved creating a strict map of expected interactions between the communication partner and the participant, along with the communication partner’s corresponding responses to those interactions (see Fig. 5 for a conceptualization of the gaze-contingent experimental setup for phases involving interactions between the communication partner and the participant).

#### Basic contingencies

The gaze-contingent experimental setup is designed to respond to participants’ anticipated gaze behaviors by using triggers that ensure stimuli flow seamlessly and reflect participants’ interactions in real time. In the IET-JA: the attention-getter phase requires a single fixation of ≥ 100 ms on the region of interest (ROI) without a specified timeframe; the initial phase for all stimuli (i.e., the only gaze-contingent phase for RJA stimuli) requires a single fixation of ≥ 200 ms on the actress’s Face ROI within a predefined timeframe; and IJA-Request and IJA-Comment/Reference stimuli require a single sequence of alternating gaze between the Face and Target ROIs without interruption and within a predefined timeframe. These basic contingencies establish a baseline for interpreting participants’ engagement with JA stimuli and offer a framework for addressing any deviations from expected gaze behavior (see Table 1 for a summary of basic gaze-contingent metrics, and Yoon et al. (2025b) for additional gaze metrics that can be derived and analyzed using the IET-JA battery).

**Table 1:** Summary of key gaze metrics for each JA task

Task phase	Gaze metric	Definition
RJA		
0	Fixation duration	A single fixation of ≥ 100 ms on a target ROI to trigger phase 1
1	Fixation duration	A single fixation of ≥ 200 ms on face ROI to trigger phase 2
3	Gaze alternation	A first fixation on target ROI from face ROI at the onset of cue as a measure of RJA
IJA-Request		
0	Fixation duration	A single fixation of ≥ 100 ms on a target ROI to trigger phase 1
1	Fixation duration	A single fixation of ≥ 200 ms on face ROI to trigger phase 2
3	Gaze alternation	A single gaze alternating of consecutive fixations between face and tablet (requested show) ROIs to trigger phase 4 as a measure of IJA request
3	Latency	Total time (ms) taken to trigger phase 4 (i.e., time until successful gaze alternating ended) as a measure of time taken to IJA-request
IJA-Comment/Reference		
0	Fixation duration	A single fixation of ≥ 100 ms on a target ROI to trigger phase 1
1	Fixation duration	A single fixation of ≥ 200 ms on face ROI to trigger phase 2
2	Gaze alternation	A single gaze alternating of consecutive fixations between face and object (unexpectedly appeared object) ROIs to trigger phase 3 as a measure of IJA-comment/reference
2	Latency	Total time (ms) taken to trigger phase 3 (i.e., time until successful gaze alternating ended) as a measure of time taken to IJA-comment/reference

*Note*. See Yoon et al. (2025b) for additional gaze metrics that can be derived and analyzed with the IET-JA battery.

#### Ensuring contingency fidelity to construct

To measure participant gaze behaviors described in the previous section with precision, gaze-contingent configurations were implemented to enforce fidelity to the experimental constructs. For example, during the initial phase of all stimuli, which requires a single fixation of ≥ 200 ms on the Face ROI, the phase does not progress (i.e., the communication partner does not respond) if a fixation of < 200 ms is on the Face ROI or if it falls outside the Face ROI. Additionally, IJA tasks require participants to alternate gaze between the Face and Target ROIs in a continuous, uninterrupted sequence. A critical aspect of this configuration is its sensitivity to interruptions. For example, if a participant’s gaze deviates outside the Target ROI during the sequence (e.g., fixation on the actress’s Face ROI followed by fixation on her hand rather than on the Target ROI), the interaction map is configured to reset the sequence. This mechanism loops back to the starting condition while the stimuli continue to play, ensuring that the intended sequence is strictly adhered to without prematurely terminating the stimuli. By implementing these configurations, the experiment preserves its ability to capture meaningful data aligned with its construct validity.

#### Handling unexpected events

##### **Contingency timeouts**

To account for situations where participants do not exhibit the expected gaze behaviors within a predefined timeframe, “timeout” triggers were implemented. These triggers ensure that the experiment continues to progress even in the absence of data or when unexpected gaze behaviors occur. For example, during the gaze-contingent phase of IJA-Request stimuli, if a participant does not exhibit the required gaze behavior (i.e., a single uninterrupted sequence of alternating gaze between the Face and Tablet ROIs), the respective phase will continue for the predefined duration and will automatically transition to the next phase. Similarly, if a participant does not sustain a fixation of ≥ 200 ms on the actress’s Face ROI during the initial phase of the stimuli, the phase will continue to play and advance after the predefined duration. This timeout mechanism preserves the flow of the stimuli while accommodating developmental considerations regarding wait times and stimulus durations for the pediatric age group. This approach blends experimental rigor with practical session constraints by ensuring continuity without unnecessary delays.

##### Manual overrides.

In addition to timeout configurations for events with no data or unexpected gaze behavior, unforeseen circumstances involving technical disruptions (e.g., significant gaze drift) are handled through manual overrides. “Override” triggers are implemented alongside all gaze triggers to maintain complete control over the experiment. For example, during the attention-getter phase, which relies on a single fixation ≥ 100 ms on the ROI near a screen corner, edge-effect issues may emerge as the accuracy and precision of gaze detection could be compromised near the edges of the screen. If such technical problems interfere with the stimuli, the “override” trigger allows the researcher to manually advance to the next phase. This safeguard ensures that technical disruptions do not undermine the overall flow or validity of the experiment.

#### Distinguishing between triggers

A distinction mechanism was configured for all triggers to ensure the integrity of the collected data. This setup differentiates between “true” gaze triggers, which directly reflect participants’ gaze behavior, and other triggers, such as timeouts or overrides. Additionally, it enables differentiation among different gaze triggers, including fixation triggers and gaze-alternating triggers. This distinction mechanism provides clarity when analyzing the data, ensuring that only participant-driven interactions with the stimuli are considered in the analysis, while accounting for exceptions handled by the system and/or researchers. As a result, the data would accurately reflect participants’ gaze behaviors under both ideal and non-ideal conditions.

### Test-running eye-tracking stimuli

A test run of the battery (i.e., all developed stimuli) was conducted with a graduate student with sufficient knowledge of autism, eye tracking, and JA to identify any potential technical issues. Experts then reviewed the test-run battery to evaluate its functionality and effectiveness. Errors encountered during test runs (e.g., glitches in video transitions, malfunctioning gaze triggers) that could not be resolved immediately prompted consultations with an SR technician. This consultation allowed for accurate replication and troubleshooting of the errors, which were then adjusted using the Experiment Builder and re-evaluated by experts. This process of testing, reviewing, and tuning was repeated iteratively. Each cycle aimed to address any outstanding technical errors and improve the overall design and execution of the battery. This approach continued until the battery was technically sound and ready for deployment for a pilot. This pilot was essential for identifying any unanticipated technical issues that had not been detected in previous test runs with a graduate student with sufficient knowledge of the battery’s functionality (e.g., how the implemented gaze triggers are intended to operate).

A pilot test of the battery was then conducted with a small group of young adults (*N* = 6) to identify any unanticipated technical issues and to perform additional tuning as necessary. The following adjustments were made as a result of this pilot: repositioning attention-getter ROIs, adjusting gaze-trigger threshold for the attention-getter and the initial phase of all stimuli, and incorporating a greeting phase to clarify the communication modality. The attention-getter ROIs were repositioned (i.e., farther from the screen corners) because the stimuli failed to trigger in many instances even when participants looked at the attention-getter ROI. This indicated potential edge effects, in which the eye tracker’s gaze detection accuracy and precision are compromised near the screen edges. Additionally, gaze-trigger threshold for the attention-getter phase and the initial phase of all JA stimuli were refined to 100 ms and 200 ms, respectively. This adjustment was necessary to mitigate seemingly slow response times and ensure that stimuli progressed smoothly without unnecessary delays. Lastly, we recognized the importance of establishing clear expectations for participants regarding the primary mode of interaction. To reinforce the nonverbal nature of communication with the preprogrammed communication partner, particularly for verbal individuals, we included the phrase “*I can see you but not hear you*” at the beginning of the battery. This statement was intended to provide context and clarify that the communication partner would rely solely on visual input (i.e., gaze behaviors) to interpret participant responses. The subsequent test runs, incorporating adjustments made after the pilot phase, confirmed the battery’s readiness for full-scale execution.

## Results

The final IET-JA battery comprises 32 stimuli: 16 RJA, 8 IJA-Request, and 8 IJA-Comment/Reference. The 32 stimuli are organized into four blocks, each containing eight stimuli (four RJA, two IJA-Request, and two IJA-Comment/Reference). The maximum duration of the IET-JA is approximately 8 min (approximately 2 min per block), without accounting for the attention-getter phase in each JA stimulus. However, due to the gaze-contingent nature of the battery, its duration is expected to vary with the participant’s level of engagement. In addition, to prevent biases arising from participants’ looking preferences and to ensure a balanced experimental design, the 32 stimuli are counterbalanced by direction (left/right), grouped into different sets of tasks (RJA, IJA-Request, IJA-Comment/Reference), and presented in an interleaved manner. The following sections detail a description of the stimuli for each JA skill included in the IET-JA.

### Description of joint attention stimuli in the IET-JA

#### Greeting and training

**Fig. 6:**
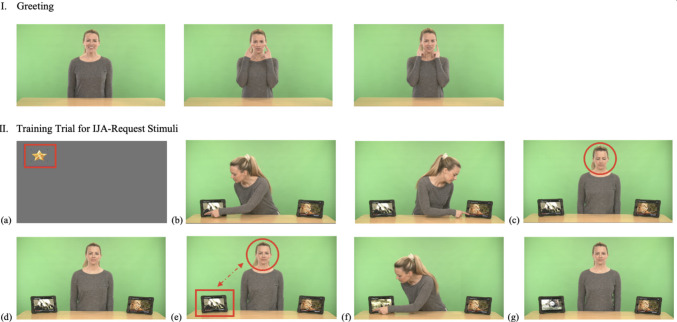
Screenshots of the greeting and training trial in the IET-JA. *Note.* Greeting: The actress uses speech and gestures to establish the mode of interaction (“*I can see you, but I cannot hear you.*”). Training trial for IJA-Request stimuli: **(a)** An attention-getter to attune the participant’s attention to the screen and mark the beginning of the stimuli; **(b)** the initial phase where the actress begins the training trial by playing videos on each of the tablets (i.e., iPads), which is not part of the actual IJA-Request stimuli; **(c)** Phase 1, the actress looks down, waiting for initial attention; **(d)** Phase 2, the actress provides opportunity to request (“*Yes?*”); **(e)** Phase 3, the actress waits for the participant to request by alternating gaze between one of the two tablets and the actress’s face; **(f)** Phase 4, the actress responds (“*Sure, I can play it for you!*”) and plays the requested video; and **(g)** Phase 5, the requested video plays. *Red outlines* (i.e., arrow, circle, rectangle) indicate ROIs and gaze behaviors defined for gaze triggers (i.e., fixation on the attention-getter, fixation on the face, gaze alternating between the face and a tablet).

The battery begins with a greeting phase (10 s), during which the actress greets the participant and provides the necessary instructions. She informs the participant that she can see them but cannot hear them, establishing the communication parameters for the task. Following the greeting, a training trial of the IJA-Request task (max 52 s) is administered to familiarize participants with the task’s expectations. During this training trial, the actress sought to convey that the videos featured on the tablets (i.e., iPads) would activate and play in response to their communication act (i.e., request). To achieve this, the actress begins the training trial by playing short videos on two tablets, one at a time, which are not part of the actual IJA-Request stimuli. This approach helps participants associate the tablets with the task and understand the tablets’ role in the subsequent IJA-Request task (see Fig. 6 for screenshots of the greeting and training trial).

#### Attention-getter

An attention-getter (i.e., a blank gray screen with an attention-getter, such as a star or balloons; see Fig. 6) is presented before each stimulus, including the training trial, to ensure that participants attend to the screen before the stimulus begins. These attention-getters are also counterbalanced by direction (left/right) to maintain consistency. Participants are required to look at the attention-getter for ≥ 100 ms to trigger each stimulus. This threshold was established through test runs and pilot sessions to ensure a smooth start to stimuli without experiencing delays.

#### RJA stimuli

The IET-JA includes 16 RJA stimuli across cues (gaze shift, head-turning) and directions (left, right). Each combination consists of four stimuli: a leftward gaze-shift cue, a rightward gaze-shift cue, a leftward head-turn cue, and a rightward head-turn cue.

**Fig. 7:**
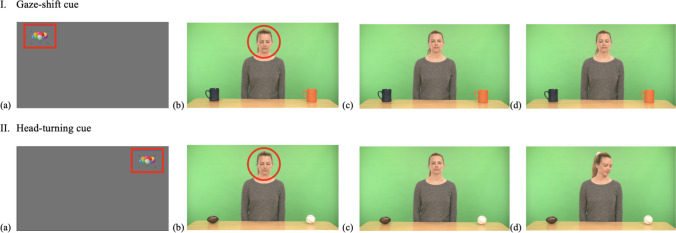
Screenshots of RJA stimuli in the IET-JA. *Note.* **(a)** An attention-getter to attune the participant’s attention to the screen and mark the beginning of the stimuli; **(b)** Phase 1, the actress looks down, waiting for initial attention; **(c)** Phase 2, the actress looks up in response to initial attention; and **(d)** Phase 3, the actress provides a bid for JA. *Red outlines* (i.e., circle, rectangle) indicate ROIs and gaze behaviors defined for gaze triggers (i.e., fixation on the attention-getter, fixation on the face).

Each stimulus consists of three phases: (a) *Phase 1*, where the actress looks down until the participant looks at her face for ≥ 200 ms within the predefined timeframe (4 s), establishing mutual initial attention and triggering Phase 2; (b) *Phase 2*, where the actress looks up and establishes direct gaze with the participant in response to their initial attention (1.5 s); and (c) *Phase 3*, where the actress provides a referential cue (gaze shift or head turn) to one of two objects positioned at the left/right corners of the table (4 s). While the maximum duration of an RJA stimulus is 9.5 s, it varies according to the participant’s level of engagement due to the gaze-contingent nature of the battery. Additionally, RJA stimuli include four different pairs of objects—two cups, two sunglasses, two hats, and two balls—systematically varied across the 16 stimuli, with each pair appearing in four stimuli (see Fig. 7 for screenshots of RJA stimuli in the IET-JA, and Supplementary Table 1 for additional descriptions of RJA stimuli parameters and features).

#### IJA to request stimuli

The IET-JA includes eight IJA-Request stimuli across directions (left, right), with four stimuli featuring a short video A on the left and a short video B on the right, and four stimuli featuring a short video A on the right and a short video B on the left.

**Fig. 8:**
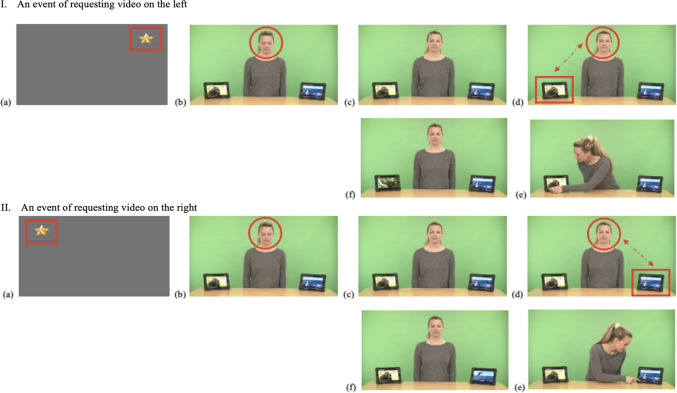
Screenshots of IJA-Request stimuli in the IET-JA. *Note.* **(a)** An attention-getter to attune the participant’s attention to the screen and mark the beginning of the stimuli; **(b)** Phase 1, the actress looks down, waiting for initial attention; **(c)** Phase 2, the actress provides an opportunity to request (“*Yes?*”); **(d)** Phase 3, the actress waits for the participant to request; **(e)** Phase 4, the actress responds (“*Sure, I can play it for you!*”) and plays the requested video; and **(f)** Phase 5, the requested video plays. *Red outlines* (i.e., arrow, circle, rectangle) indicate ROIs and gaze behaviors defined for gaze triggers (i.e., fixation on the face, alternating gaze between the face and a tablet).

Each stimulus consists of five phases: (a) *Phase 1*, where the actress looks down until the participant looks at her face for ≥ 200 ms within the predefined timeframe (4 s), establishing mutual initial attention and triggering Phase 2; (b) *Phase 2*, where the actress looks up and provides an opportunity for the participant to request one of the two short videos displayed on the tablets by saying “*Yes?*” while maintaining direct gaze with the participant (1.5 s); (c) *Phase 3*, where the actress maintains direct gaze with the participant, while waiting for them to request their choice by alternating gaze between a tablet (left or right) and the actress’ face within the predefined timeframe (7 s), subsequently triggering Phase 4; (d) *Phase 4*, where the actress responds to the participant’s request using speech (i.e., “*Sure, I can play it for you!*”) and gestures (i.e., pressing the play button of the short video displayed on the tablet; 3.5 s); and (e) *Phase 5*, where the actress maintains direct gaze (i.e., not attending to the short video that is playing) while the requested short video plays for the participant to watch (10 s). While the maximum duration of an IJA-Request stimulus is 26 s, it varies according to the participant’s level of engagement. Additionally, IJA-Request stimuli include eight short videos on YouTube, presented in pairs and systematically varied across the eight stimuli, with each pair appearing in two stimuli (see Fig. 8 for screenshots of IJA-Request stimuli in the IET-JA, and Supplementary Table 1 for additional descriptions of IJA-Request stimuli parameters and features).

#### IJA to comment/reference stimuli

The IET-JA includes eight IJA-Comment/Reference stimuli across directions (left, right), with four stimuli featuring a moving object appearing on the left and four stimuli featuring a moving object appearing on the right.

**Fig. 9:**
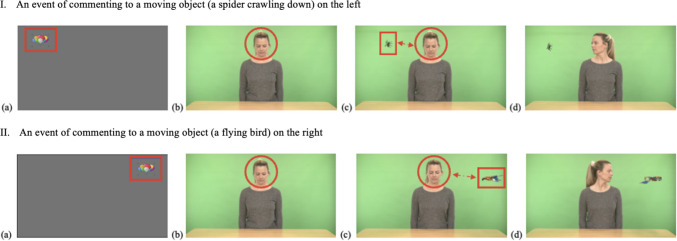
Screenshots of IJA-Comment/Reference stimuli in the IET-JA. *Note*. **(a)** An attention-getter to attune the participant’s attention to the screen and mark the beginning of the stimuli; **(b)** Phase 1, the actress looks down, waiting for initial attention; **(c)** Phase 2, the actress remains looking down, waiting for the participant to comment/reference the object (spider in I, bird in II); and **(d)** Phase 3, the actress responds to commenting/referencing. *Red outlines* (i.e., arrow, circle, rectangle) indicate ROIs and gaze behaviors defined for gaze triggers (i.e., fixation on the face, alternating gaze between the face and the object).

Each stimulus consists of three phases: (a) *Phase 1*, where the actress looks down until the participant looks at her face for ≥ 200 ms within the predefined timeframe (4 s), establishing mutual initial attention and triggering Phase 2; (b) *Phase 2*, where the actress remains looking down while an object suddenly appears in the left/right top corner of the screen, waiting for the participant to draw her attention to the moving object by alternating gaze between the moving object and the actress’ face within the predefined timeframe (7 s), subsequently triggering Phase 3; and (c) *Phase 3*, where the actress responds to the participant’s commenting/referencing using speech and gesture simultaneously (i.e., “*Oh!*” while turning her head to look at the object; 3 s). While the maximum duration of an IJA-Comment/Reference stimulus is 14 s, it varies according to the participant’s level of engagement due to the gaze-contingent nature of the battery. Additionally, IJA-Comment/Reference stimuli include four different moving objects—a spider crawling down, a parrot flying in, a ball swinging, and a snake crawling down—systematically varied across the eight stimuli, with each object appearing in two stimuli (see Fig. 9 for screenshots of IJA-Comment/Reference stimuli in the IET-JA, and Supplementary Table 1 for additional descriptions of IJA-Comment/Reference stimuli parameters and features).

## Discussion

This study aimed to develop a gaze-contingent eye-tracking battery focused on three developmentally appropriate JA skills for young children: RJA, IJA to request, and IJA to comment or reference. The newly developed IET-JA battery extends previously employed eye-tracking paradigms by integrating gaze contingency and including more comprehensive JA constructs.

Despite the inherently interactive nature of JA, many studies have often relied on passive paradigms to study JA in young autistic children (Yoon et al., 2026). The key characteristic that makes the newly developed IET-JA critical for JA research in this population is its establishment of the communication partner featured in stimuli as a responsive entity, achieved through gaze-contingency integration and fostering mutual engagement. This design allows stimuli to interact dynamically with the participant’s eye movements, creating more realistic and natural JA scenarios. By incorporating gaze contingency, the battery mirrors more authentic social interactions and highlights the reciprocal, bidirectional nature of JA (Mundy & Bullen, 2022).

Including stimuli that assess both functions of IJA—to request (protoimperative) and to comment/reference (protodeclarative)—alongside RJA addresses a significant gap in the literature. Previous eye-tracking research on JA in young autistic children has predominantly focused on RJA, often overlooking the importance of IJA, despite compelling evidence suggesting that the spontaneous nature of IJA bears a more profound significance in core characteristics associated with autism than the reflexive and obligatory nature of RJA (Jones & Carr, 2004; Mundy & Newell, 2007). Furthermore, while some studies have examined IJA, the two functions of IJA, requesting versus commenting/referencing, were often not distinguished, which is essential for understanding the different social motivations behind these behaviors. By incorporating both functions of IJA and more clearly delineating the contexts for requesting and commenting/referencing behaviors, the IET-JA offers a more comprehensive approach to studying the full spectrum of JA. This more refined approach could provide greater insights into how autistic children engage in JA using gaze, potentially contributing to a more nuanced understanding of the atypical development of social communication.

While initially designed for young children, the battery is versatile and can be adapted to different age groups, developmental profiles, and study objectives within autism research. For example, the consistent use of a green background across all stimuli enables the integration of diverse virtual elements or settings, such as animated backgrounds, to meet the needs of various studies. Similarly, using tablets with green screens in IJA-Request stimuli enables researchers to superimpose a range of digital content, such as age-appropriate cartoons or even photographs, to match participants’ developmental levels. For RJA and IJA-Comment/Reference stimuli, raw video footage devoid of target objects in the scene provides the flexibility to insert different objects, actions, or graphic elements in post-production, ensuring that stimuli can be tailored to specific research questions. This adaptability highlights IET-JA’s value as a customizable tool with the potential to address current gaps in JA research and meet the evolving needs of researchers, thereby broadening its applicability across a range of research contexts and goals.

Lastly, the development of the IET-JA highlights the essential role of team science and an iterative, feedback-driven approach (Kern et al., 2011; Wynn & Eckert, 2017). This process involved designing JA stimuli aligned with early developmental milestones, multiple rounds of prototyping, video recording and editing, and testing procedures. Experts from various fields reviewed each stage, providing valuable feedback that led to continual refinement, ensuring the battery’s developmental appropriateness, ecological validity, and methodological rigor. Through this iterative process of feedback, revision, and improvement, the battery was designed to meet diverse research needs while remaining flexible and rigorous. The collaboration brought together the expertise of a behavior analyst, computer scientist, digital specialist, psycholinguist, developmental scientist, and special educator, each contributing to the development of the battery at every step. This multidisciplinary effort drove innovation and reinforced the importance of integrating perspectives from various fields to tackle the complex challenges of autism research (National Research Council, 2015).

### Limitations and future directions

While the IET-JA offers potential benefits for studying JA in young autistic children during interactive episodes, several limitations should be acknowledged. A significant limitation is the lack of validation data for the targeted pediatric population. Previous eye-tracking research involving tasks similar to the IET-JA, but without gaze contingency, has shown that JA outcomes in young autistic children differ significantly from those of typically developing peers and are linked to the severity of autistic symptoms (e.g., Billeci et al., 2016; de Belen et al., 2023; Muratori et al., 2019; Zhang et al., 2022), behavioral measures of JA (Franchini et al., 2017) and adaptive functioning (Falck-Ytter et al., 2012; Muratori et al., 2019) in the expected direction. Additionally, a few studies incorporating gaze contingency have also been successful. Specifically, Liu et al. (2024), using a virtual game format for RJA with an interactive virtual character, found that different cues significantly impact the RJA performance of autistic children, with no significant effect on typically developing peers; Wang, Hoi et al. (2020) examined gaze responses of autistic children to a virtual character’s gaze following in a reciprocal JA context and found that autistic children, but not typically developing peers, responded less effectively to the virtual character’s gaze following their own gaze. Moreover, Wang, Wall et al. (2020) assessed the feasibility of automated gaze-modification training to improve social attention in 3-year-old autistic children and found that gaze-contingent training effectively mitigated decreases in attention toward the faces of on-screen social characters in autism. Notably, a previous study applying the IET-JA in young adults (Yoon et al., 2025b) found significant associations between IET-JA measures and autistic symptoms, providing promising preliminary evidence of construct validity. Consequently, these prior studies—both with and without gaze contingency—collectively suggest that the primary issue is not feasibility or the related constructs under investigation but whether the IET-JA substantially improves ecological validity, psychometric robustness and the strength of associations between eye tracking and behaviorally measured JA. While we acknowledge that these results cannot replace the validation of the IET-JA in children, which is currently underway, the design of construct-appropriate content for such gaze-contingent systems is likely universal and of broad interest. This is especially true considering the limited knowledge and lack of formalization regarding critical decision points in gaze-contingent construction around behavioral targets, and many pitfalls and considerations would remain unclear without guidance. For example, this development outlines fallback strategies for handling various situations, including manual decision points as a last resort; categorizes JA constructs into distinct profiles of investigation; considers critical video recording and technical factors (e.g., lighting, audio quality, output consistency, background discrepancies) to ensure smooth and cohesive stitching of raw video segments, which is vital for gaze contingency; and defines the start and end points of video filming to create a seamless interactive experience for end users. Therefore, this work should be viewed as a developmental step that lays the groundwork for future validation studies in pediatric populations, which are currently underway and essential for evaluating the reliability, validity, and clinical utility of the IET-JA. Second, the battery involves only a single communication partner, a White female adult. This homogeneity might unintentionally introduce biases or limitations when interpreting participant responses in JA interactions, as individuals from diverse backgrounds may perceive and engage with a communication partner differently due to cultural, social, or linguistic factors. For example, children from non-White or non-Western cultural backgrounds may respond differently or feel less comfortable when interacting with a communication partner who does not share their sex, culture, or ethnicity (Blais et al., 2008; Ma et al., 2021). Although using a female adult as the communication partner is common in eye-tracking research examining JA in young autistic children (e.g., Billeci et al., 2016; Cilia et al., 2022; de Belen et al., 2023; Falck-Ytter et al., 2012), future studies should include more diversity in communication partners to improve the ecological validity and cultural relevance of the battery across diverse populations with autism. Third, when using the battery with children, particular care must be taken to ensure the content is appropriate and comfortable for them. Institutional Review Boards and researchers should consider whether certain objects (e.g., animals like spiders and snakes) may elicit fear and consult with parents or guardians regarding their acceptability. While the IET-JA’s design allows for flexibility in replacing or modifying stimuli, such changes should be re-evaluated psychometrically to confirm that measurement validity and reliability are maintained. Fourth, the selection of JA tasks included in the battery was constrained by the capabilities of eye-tracking technology, which relies solely on the participant’s gaze as the primary mode of communication. As a result, some contextual aspects of live JA interactions—such as spontaneous verbalizations or reciprocal gestures like pointing—cannot be considered in this work. Although gaze is a useful and measurable proxy for JA, relying on it alone risks oversimplifying the phenomenon, as it does not capture the multimodal nature of JA in real-world settings like home or school. Future development of the battery should aim to incorporate additional behavioral channels to better reflect the complexity and variability of natural JA interactions. Lastly, technical challenges related to the IET-JA’s gaze-contingent design may occur. For example, issues like significant gaze estimation errors arising from drift between initial calibration and validation points could disrupt stimulus triggering, as observed during the pilot stage. While we implemented ‘Manual override’ and ‘contingency timeout’ functions to address these issues, related errors can still interfere with the battery’s progress and the quality of the collected data. Although we chose not to include a function for repeating failed trials due to concerns about attentional demands and the need to maintain a clear session endpoint, future versions of the IET-JA could consider adding such a feature. A controlled repetition process might help recover more valid trials and increase data yield, while balancing participant burden with careful limits and timing. Therefore, ongoing efforts to improve the battery’s technical accuracy and robustness are crucial for ensuring the integrity of findings derived from its use.

### Implications

The development of the IET-JA has several implications for future autism research. One key implication is the advancement in methodological design. Adopting the gaze-contingent paradigm represents a shift toward more ecologically valid eye-tracking experiments for studying JA in pediatric populations. As previously mentioned, eye-tracking research on JA in young autistic children has often relied on passive paradigms, which may limit the ability to capture naturalistic, real-life JA interactions. In contrast, the gaze-contingent approach adjusts stimuli in response to the participant’s gaze behavior, creating a more interactive eye-tracking experience during JA tasks. While it is not without limitations, this methodological approach offers a complementary method for studying JA in contexts that more closely mimic real-world social interactions. Another implication is the demonstration of multidisciplinary collaboration. Engaging experts from different disciplines, such as behavioral science, computer science, digital media and psychology, was crucial in developing the IET-JA. This collaborative approach ensures the tool is scientifically rigorous and practically relevant. For example, expertise in computer science from a biomedical research context enabled the development of the gaze-contingent paradigm, aligning its technical feasibility with the social constructs under investigation. Insights from behavioral science informed the design of JA stimuli to reflect the social dynamics inherent to JA, ensuring that the tool maintained ecological validity. This collaboration can serve as an example for future research, illustrating how diverse expertise can be leveraged to develop tools capable of addressing complex research questions. Finally, the iterative process of designing, prototyping, testing, and refining the IET-JA highlights the value of flexibility and responsiveness in research. Each development cycle was informed by detailed feedback from experts across various disciplines, which helped identify areas for improvement and ensured that the final product met the standards of accuracy and reliability. This iterative approach could be applied to other areas of autism research, promoting a culture of continuous refinement and innovation. In summary, developing the IET-JA offers valuable insights for advancing methodologies, promoting team science, and implementing iterative processes in autism research.

## Supplementary information

Below is the link to the electronic supplementary material.

Supplementary file1 (DOCX 2985 KB)

## Data Availability

Data availability not applicable. Sample videos included in the IET-JA can be accessed here: https://github.com/cyoon37/Interactive-Eye-tracking-Battery-for-Joint-Attention.

## Code availability

SR-specific implementation maps for the stimuli included in the IET-JA can be accessed here: https://github.com/cyoon37/Interactive-Eye-tracking-Battery-for-Joint-Attention.

## Declarations

### Copy rights

The authors have the rights to the IET-JA.
