# Supplementary material for: Towards the ecological automated measurement of joint attention: Development of an interactive eye-tracking battery for joint attention in children with and without autism
Source: Behav Res Methods. 2026 May 13;58(6):167. doi: 10.3758/s13428-026-03017-w (PMC13171993; doi:10.3758/s13428-026-03017-w)
Supplement: Supplementary file 1 — Supplementary file1 (DOCX 2985 KB) [file 13428_2026_3017_MOESM1_ESM.docx]

|  | Phase | Actress’s Communicative Cue | Actress’s Act | Gaze-contingency | Duration  (sec) |
| --- | --- | --- | --- | --- | --- |
| RJA Stimuli | | | | | |
|  | 1 | none | looking down | a single fixation of ≥ 200 ms on face | 4 (max) |
|  | 2 | direct gaze | looking up | no | 1.5 |
|  | 3 | gaze shift or head-turn | providing bid for JA | no | 4 |
| Total | 3 phases |  |  | **1 gaze-contingent phase** | **9.5 sec*** |
| IJA-Request Stimuli | | | | | |
|  | 1 | none | looking down | a single fixation of ≥ 200 ms on face | 4 (max) |
|  | 2 | direct gaze + direct speech | looking up and providing an opportunity to request, saying, “*Yes?*” | no | 1.5 |
|  | 3 | direct gaze | waiting for a request | a single alternating gaze between face and iPad (without interruption) | 7 (max) |
|  | 4 | direct gaze + direct speech | playing the requested video, saying “*Sure, I can play it for you!*” | no | 3.5 |
|  | 5 | direct gaze | looking at the ‘camera’ while the requested video is playing | no | 10 |
| Total | 5 phases |  |  | **2 gaze-contingent phases** | **26 sec*** |
| IJA-Comment/Reference Stimuli | | | | | |
|  | 1 | none | looking down | a single fixation of ≥ 200 ms on face | 4 (max) |
|  | 2 | none | looking down during the appearance of an object, waiting for a comment | a single alternating gaze between face and object (without interruption) | 7 (max) |
|  | 3 | direct gaze + head turn + direct speech | turning to look at the object and saying, “*Oh!*” | no | 3 |
| Total | 3 phases |  |  | **2 gaze-contingent phases** | **14 sec*** |

**Supplementary Table 1.** Description of JA stimuli parameters and features.

*Note*. * Expected duration since stimuli involve gaze-contingent phases, which depend on the participant’s level of engagement. For phases that are gaze-contingent, the indicated duration is the timeframe (i.e., max phase duration) during which the participant can use their gaze to trigger the respective phase. JA = joint attention; IJA = initiating joint attention; RJA = responding to joint attention
